# Detection of *Toxoplasma gondii* oocysts in soils in Northwestern China using a new semi-nested PCR assay

**DOI:** 10.1186/s12917-014-0238-z

**Published:** 2014-09-28

**Authors:** Meng Wang, Peng Meng, Qiang Ye, Yuan-Hua Pu, Xiao-Yu Yang, Jian-Xun Luo, Nian-Zhang Zhang, De-Lin Zhang

**Affiliations:** State Key Laboratory of Veterinary Etiological Biology, Key Laboratory of Veterinary Public Health of the Ministry of Agriculture, Key Laboratory of Veterinary Parasitology of Gansu Province, Lanzhou Veterinary Research Institute, Chinese Academy of Agriculture Sciences, Lanzhou, Gansu Province 730046 PR China

**Keywords:** *Toxoplasma gondii*, Oocysts, Semi-nested PCR, Soil

## Abstract

**Background:**

*Toxoplasma gondii* is a zoonotic pathogen that can infect a range of animals and humans. Ingestion of *T. gondii* oocysts in soil is a significant transmission route for humans and animals acquiring toxoplasmosis*.* In the present study, we developed a new semi-nested PCR method to determine *T. gondii* oocysts distribution in soils in northwestern China.

**Results:**

The one tube semi-nested PCR assay was developed to detect the oocysts of *T. gondii* in soil, targeting the repetitive 529 bp fragment of *T. gondii* genomic DNA. Then a total of 268 soil samples, including 148 samples from Gansu Province and 120 samples from Qinghai Province, northwestern China, were examined by the semi-nested PCR method. One third of the positive samples were sequenced. The sensitivity of the semi-nested PCR assay was 10^2^ *T. gondii* oocysts in 5 g soil sample. Investigation of soil samples from northwestern China showed that 34 out of 268 soil samples (12.69%) were *T. gondii* positive. Sequences of the partial 529 bp fragments varied from 0–1.2% among the sequenced samples. The prevalence of *T. gondii* oocysts in soil from cities (24/163) was slightly higher than that in soils from pasturing areas (10/105) (*P* = 0.21). Among the different regions in cities, the prevalence of *T. gondii* oocysts in soils from parks was 14.15%, whereas that in soils from schools was 19.05%.

**Conclusions:**

The present study firstly reported the prevalence of *T. gondii* oocysts in soils in northwest China using a novel semi-nested PCR assay, which provided baseline data for the effective prevention and control of toxoplasmosis in this region.

## Background

Toxoplasmosis caused by *Toxoplasma gondii* is a zoonotic infection of human beings and animals [1-5]. The parasite can lead to severe disease or even death in immunocompromised hosts such as AIDS patients, organ transplant recipients and malignancy patients and can also result in abortion, stillbirth or other serious consequences in newborns when the infection occurs in pregnant women [1,6,7]. *T. gondii* infection in livestock usually causes abortions, stillbirths, and neonatal deaths, especially in sheep and goats, which have been leading to serious economic losses worldwide [1,4].

The sporulated oocysts of *T. gondii* are resistant to harsh climatic circumstances [1,4]. The moist condition can prolong the survival time to more than a year. Ingestion of soil, food or water contaminated by sporulated *T. gondii* oocysts after shedding from felids is considered significant routes of *T. gondii* transmission to humans and animals [8]. Infections through oocysts have been widely reported in China and many other countries [7,9-11]. Thereinto, exposure to contaminated soil is identified as a strong risk factor revealed by studies from Europe and USA, particularly high for children [12-14]. The contaminated soil may also transfer oocysts to vegetables and fruits for human consumption, which increase risks of primary infection [15,16].

Due to the important role of contamination of soil in the transmission dynamics of *T. gondii*, previous studies developed several methods such as loop-mediated isothermal amplification (LAMP) and QT-PCR to investigate its oocysts in soils [17-20]. However, no information about the *T. gondii* oocysts prevalence in soils could be available in northwestern China, especially in the pasturing areas. The objective of the present study was to investigate prevalence of *T. gondii* oocysts in the soil samples from Qinghai and Gansu provinces in northwest China. Due to the low concentration of *T. gondii* contained in soils, we developed a new semi-PCR method based on the 529-bp repeat element to improve the sensitivity.

## Methods

### Ethics statement

This study was approved by the Ethics Committee of Lanzhou Veterinary Research Institute, Chinese Academy of Agricultural Sciences (Approval No. LVRIAEC2012-018), and the soil samples were collected strictly according to the requirements of the Ethics Procedures and Guidelines of the People’s Republic of China.

### The investigated site

The present study was carried out in Qinghai province (31°-39°N, 88°-103°E) and Gansu province (34.6°-36.73°N, 101.94°-108.43°), which lie on the northwestern of People’s Republic of China. The average elevation of Qinghai province is 3 000 m above sea level, and Gansu province is more than 1 000 m above sea level. The annual precipitation of the survey regions are below 500 mm and average annual temperature are between −4.3°C and 14.8°C. The survey regions belong to typical continental climate.

148 soil samples from 5 schools and 2 parks were collected in Lanzhou city, and the other 120 samples were collected from Huzhu, Ping’an, Huangyuan, Xining, Haibei and Hainan cities in Qinghai Province. Thereinto, Xining and Lanzhou cities are capitals of Qinghai and Gansu Provinces, respectively, and the rest 5 cities locate in the pasturing areas.

### *T. gondii* oocysts cultivation and DNA extraction

Three captive-bred cats (three-month old) were inoculated *T. gondii* GJS strain orally via a stomach tube. One week after the challenge, the *T. gondii* oocysts were isolated from feces and were purified using differential centrifugation according to Haydee et al. [18]. The sediments were kept in 2% sulfuric acid at room temperature to form sporulated oocysts, and then stored at 4°C until further analysis. The genomic DNA from soil samples were extracted using the stool DNA kit (Omega Bio-Tek Inc, USA) according to the manufacturer’s instructions.

### Development of the semi-nested PCR

To detect *T. gondii* oocysts, the specific semi-nested PCR reaction was developed targeting the *T. gondii* 529-bp fragment (accession number: AF146527). The length of targeting fragment to design the primers was 344 bp, with the primers of TX1: 5′-CAGGGAGGAAGACGAAAGTTG-3′, TX2: 5′-CACAGAAGGGACAGAAGT-3′, TX3: 5′-CTGTGTCACGTAGACCTAAGG-3′. The amplification mixture consisted of 12.5 μl of 2 × reaction mix (PCR Premix *Tag*, Takara), 0.004 μM of TX1, 0.2 μM of TX2, 0.2 μM of TX3, 0.5 μl of 1% BSA, and 1 μl of soil template DNA in a final volume of 25 μl. The reaction mixture was initially incubated for 3 min at 95°C to denature the template DNA, and then samples were amplified as follows: 15 cycles of denaturation at 95°C for 30 s and annealing/extension at 64°C for 40 s, extension at 72°C for 5 min, 35 cycles of denaturation at 95°C for 30 s and annealing/extension at 55°C for 40 s, extension at 72°C for 8 min. Semi-nested PCR product was subjected to electrophoresis on a 2.0% agarose gel in a Tris-acetic acid-EDTA (TAE) buffer at 80 V for 30 min and visualized under UV light after staining with ethidium bromide.

### Experiment soil sample recovery and soil sample DNA extraction

Seven oocyst-free soil samples (5 g) were prepared in a laboratory and experimentally contaminated with oocysts in the following amounts: 10^6^, 10^5^, 10^4^, 10^3^, 10^2^, 10^1^, and 10^0^. Every soil sample (including experimental and field samples) was filtered by double gauzes, and then genomic DNA of *T. gondii* from soil samples were extracted from 0.4 g soil using the soil DNA kit (Omega Bio-Tek Inc, USA) according to the manufacturer’s instructions. The DNA was eluted in 70 μl elution buffer and stored at −20°C until further analysis.

### Application of semi-nested PCR for soil sample

To evaluate the feasibility of the semi-nested PCR assay for the analysis of field samples, a total of 268 field soil samples were collected from Gansu and Qinghai provinces, China. The genomic DNA of *T. gondii* oocysts were isolated as described above. The samples were subjected to the developed semi-nested PCR to detect *T. gondii* oocysts in soil. Each sample was performed in triplicate. Ten of the positive samples were randomly collected and then sent to Sangon Biotech Co. Ltd. (China) for sequencing.

### Statistical analyses

The difference in the prevalent rates of *T. gondii* between regions in soil samples were analyzed by Chi-square analysis in SAS (Statistical Analysis System, Version 8.0). Values of *P* < 0.05 were considered as statistically significant.

## Results

**Figure 1:**
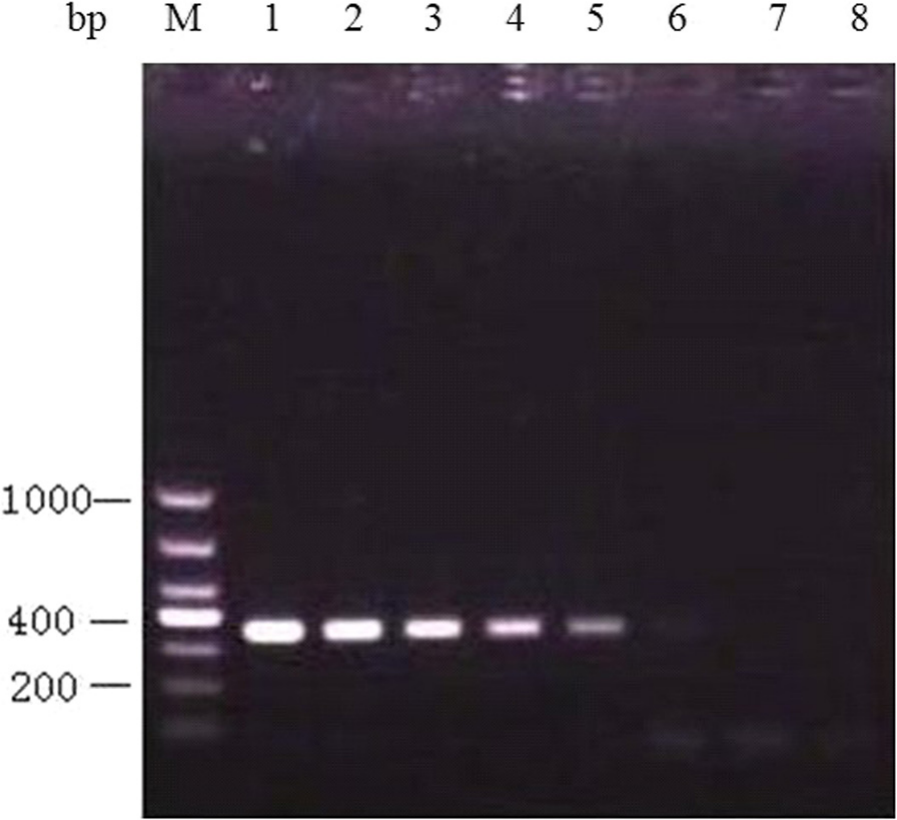
**Recovery test of** ***T. gondii*** **oocysts from experimentally contaminated soil samples.** M: DL1000 DNA Marker; line 1–7: soil samples contaminated with 10^6^-10^0^ oocysts; line 8: negative control.

The sensitive detection of the semi-nested PCR assay was determined by 10-fold serial dilution of the oocysts amount. The result showed that the method could detect the limitation of 10^2^ *T. gondii* oocysts in 5 g soil sample (Figure 1).

As shown in Table 1, 34 out of 268 soil samples (12.69%) were detected *T. gondii* oocysts positive using the semi-nested PCR method. The sequencing results indicated that the 10 amplicons were homologous to 529-fragment of *T. gondii* and a lot of 4 variable nucleotide positions were identified in the obtained sequences, ranging from 0–1.2%, which included 1 transtion (C < − > A) and 3 transversions (A < − > T and C < − > G). The prevalence of *T. gondii* oocysts in soils from cities (24/163) was higher than that from pasturing areas (10/105), and the difference was not statistically significant (*P* = 0.21). Among the different regions in Lanzhou city, the prevalence in soils from schools was 14.15% (15/106), which was statistically not significantly different to that in soils from parks (8/42, *P* = 0.66) (Table 2).

**Table 1:** **Detection of oocysts of** ***Toxoplasma gondii*** **DNA in soil samples**

**Type of area**	**Regions**	**No. examined**	**Positive**	
			**No.**	**% (95%** **CI)**
City	Lanzhou	148	23	15.54 (9.7–21.38)
	Xining	15	1	6.7 (0–19.29)
Graze area	Huzhu	20	2	10 (0–23.15)
	Pingan	10	0	0
	Huangyuan	10	0	0
	Haibei	30	4	13.33 (1.17–25.5)
	Hainan	35	4	11.43 (0.89–21.97)
Total		268	34	12.69 (8.64–16.55)

**Table 2:** **Detection of oocysts of** ***Toxoplasma gondii*** **in the soil samples in Lanzhou City**

**Areas**	**No. examined**	**Positive**	
		**No.**	**% (95%** **CI)**
School A	20	5	25 (6.02–43.98)
School B	22	2	9.09 (0–21.1)
School C	22	4	18.18 (2.07–34.3)
School D	20	2	10 (0–23.15)
School E	22	2	9.09 (0–21.1)
Park A	22	5	22.72 (5.22–40.24)
Park B	20	3	15 (0–30.65)
Total	148	23	15.54 (9.7–21.38)

## Discussion

Soil is a significant environmental source of *T. gondii* infection in humans and animals [12,13]. A previous study detected that 18 out of 101 soil samples were performed positive against *T. gondii* oocysts in environmental soil samples in Poland using PCR method based on the B1 gene [17]. In that study, the parasite contamination of the soil sample with at least 10^3^ oocysts for 40 g soil could be detected [17]. Another study showed that the detection limit of PCR method using 529-fragment was 5 tachyzoites in soil similar with that of loop-mediated isothermal amplification (LAMP) method, which the sensitivity was higher than the PCR method using B1 gene [13,18]. Edvinsson et al. [19] also detected the 529 bp repeat element gave better sensitivity than the B1 gene at low concentrations of *T. gondii* DNA. Thus, the 529 bp repeat element was usually used as target molecular to develop detection method of *T. gondii* [18-24]. In the present study, we developed a new semi-nested PCR method to detect *T. gondii* oocysts in soils based on the 529 bp repeat fragment, which was more sensitive than the PCR amplification of B1 gene [17]. To ensure the validity of our investigation, one third of the positive samples were chosen to sequence.

A total of 268 soil samples from northwestern China were further applied in epidemiological studies with the semi-nested PCR method. Positive DNA against *T. gondii* oocysts was detected in 34 (12.69%, 95% CI 8.64-16.55) soil samples. The prevalence of oocysts in soil was lower than that in Hubei province, China by PCR method and in São Paulo, Brazil by immunohistochemistry and indirect fluorescent antibody test (IFAT) [19,22]. The different positive rates in these studies may be due to differences in diagnostic methods used, animal welfare especially the care of cats, as well as the different environments. The investigated places belong to the typical continental monsoon climate with the dry and cold circumstances. Furthermore, the UV radiance in these plateau regions are little stronger than that in the flat areas. These environmental conditions were harsh for the survival of *T. gondii* oocysts, and were unfavorable for epidemics of toxoplasmosis [25,26], which was coincided with previous studies that seroprevalence of the parasite in pet animals [27] and poultry [28] in Lanzhou city and in free-grazed animals [29] was lower than that in most of other moist places.

City was >1 × more than pasturing area to be contaminated *T. gondii* oocysts (OR 1.64, 95% CI 0.75-3.59). The widespread distribution of *T. gondii* in city was mainly caused by stray or free-living cats. In our previous study, we isolated 11 *T. gondii* strains from 41 stray cats [27], therefore, ubiquitous stray cats infected by *T. gondii* are contributed to the high prevalence of the parasite and constant infection pressure in the environment [8]. We can also find support for this tendency from the higher prevalence in soil samples from school. The school A and school C were not far from pet animal markets. The residual foods on the ground attract stray cats to eat. The school A and C may become the haunt of stray cats.

The *T. gondii* prevalence in schools indicates a main risk source of infection for younger, especially the presence in soil from school C and E. The school C and E were elementary schools. Students, sometimes, may play with earth. They may be infected with *T. gondii* due to blot hands with the oocysts contaminated soil and then eat food without any awareness. So, parents should be take appropriate precautions. In addition, regularly cleaning the surroundings and other integrated strategies and measures are necessary for the effective prevention and control of *T. gondii* oocysts prevalence.

## Conclusion

The present study developed a new semi-nested PCR assay based on the repetitive 529 bp fragment of *T. gondii* genomic DNA. Using this assay, high level (12.69%) contamination of *T. gondii* oocysts in soils in northwestern China was revealed for the first time, indicating a potential risk of soil as an important source of *T. gondii* infection in humans and other animals in this region of China.
